# Deep Sequencing of Norovirus Genomes Defines Evolutionary Patterns in an Urban Tropical Setting

**DOI:** 10.1128/JVI.01333-14

**Published:** 2014-10

**Authors:** Matthew Cotten, Velislava Petrova, My V. T. Phan, Maia A. Rabaa, Simon J. Watson, Swee Hoe Ong, Paul Kellam, Stephen Baker

**Affiliations:** a The Wellcome Trust Sanger Institute, Hinxton, United Kingdom; b Hospital for Tropical Diseases, Wellcome Trust Major Overseas Programme, Oxford University Clinical Research Unit, Ho Chi Minh City, Vietnam; c Centre for Immunity, Infection and Evolution, University of Edinburgh, Edinburgh, United Kingdom; d Division of Infection & Immunity, University College London, London, United Kingdom; e The London School of Hygiene and Tropical Medicine, London, United Kingdom; f Centre for Tropical Medicine, University of Oxford, Oxford, United Kingdom

## Abstract

Norovirus is a highly transmissible infectious agent that causes epidemic gastroenteritis in susceptible children and adults. Norovirus infections can be severe and can be initiated from an exceptionally small number of viral particles. Detailed genome sequence data are useful for tracking norovirus transmission and evolution. To address this need, we have developed a whole-genome deep-sequencing method that generates entire genome sequences from small amounts of clinical specimens. This novel approach employs an algorithm for reverse transcription and PCR amplification primer design using all of the publically available norovirus sequence data. Deep sequencing and *de novo* assembly were used to generate norovirus genomes from a large set of diarrheal patients attending three hospitals in Ho Chi Minh City, Vietnam, over a 2.5-year period. Positive-selection analysis and direct examination of protein changes in the virus over time identified codons in the regions encoding proteins VP1, p48 (NS1-2), and p22 (NS4) under positive selection and expands the known targets of norovirus evolutionary pressure.

**IMPORTANCE** The high transmissibility and rapid evolutionary rate of norovirus, combined with a short-lived host immune responses, are thought to be the reasons why the virus causes the majority of pediatric viral diarrhea cases. The evolutionary patterns of this RNA virus have been described in detail for only a portion of the virus genome and never for a virus from a detailed urban tropical setting. We provide a detailed sequence description of the noroviruses circulating in three Ho Chi Minh City hospitals over a 2.5-year period. This study identified patterns of virus change in known sites of host immune response and identified three additional regions of the virus genome under selection that were not previously recognized. In addition, the method described here provides a robust full-genome sequencing platform for community-based virus surveillance.

## INTRODUCTION

Norovirus is a nonenveloped, positive-sense, single-stranded RNA virus approximately 7.5 to 7.7 kb in length (reviewed in reference 1). The viral genome is organized into three (or four in the case of murine norovirus [MNV] [2]) open reading frames (ORFs) that encode several structural and nonstructural proteins. ORF1 encodes a large polyprotein that is proteolytically cleaved into six nonstructural proteins, including the N-terminal p48 protein (NS1-2), an NTPase (NS3), the 3A-like p22 protein (NS4), the viral genome-linked VpG protein (NS5), the 3C-like protease 3CLpro (NS6), and the RNA-dependent RNA polymerase RdRp (NS7). Note that the nomenclature for the NS proteins is currently in flux, and both existing names have been included (3). ORF2 overlaps ORF1 by a short region and encodes the major capsid protein VP1, comprising an S (shell) domain connecting the two P (protruding) subdomains, P1 and P2, with the P2 domain binding to histo-blood group antigens (HBGAs) on target host cells. ORF3, located at the 3′ end of the genome, encodes the minor capsid protein VP2.

Norovirus is one of the genera in the Caliciviridae family of viruses and can be further classified into different genogroups (reviewed in reference 1). Noroviruses are known to cause diseases in humans (genogroups GI, GII, and GIV) and a number of other mammals and include porcine (GII), ovine/bovine (GIII), canine (GIV), and murine (MNV, forming the distinct genogroup GV) viruses (4–12).

In humans, norovirus is a highly infectious pathogen that causes a severe gastrointestinal disease in susceptible individuals after the ingestion of an exceptionally small number of viral particles. The virus is so infectious that the probability of symptomatic disease from a single norovirus virion has been estimated to be as high as 0.5 (13). The dose required to infect 50% of test subjects has been estimated to be 1,000 to 3,000 virus genome equivalents (14). A typical norovirus infection can result in profuse volumes of feces and vomitus containing 10^6^ to 10^9^ stable, nonenveloped virions per milliliter of excreta, creating almost infinite opportunities for onward transmission and additional infections. An inability to culture human noroviruses in a laboratory prevents the testing of inactivation and disinfection methods and further complicates control efforts. These issues highlight some of the difficulties in eliminating infectious norovirus from food supplies and the environment and indicate the need for the development of intelligent approaches to prevent norovirus transmission and infection.

An effective approach to controlling norovirus may be to understand how norovirus evades the human immune system and use this information to develop novel therapeutic options. Norovirus infection in a “healthy” individual is typically short and self-limiting, which results in transient or short-lived immunity (15, 16). No approved drugs that block virus replication exist. Accordingly, public health measures to identify and eliminate sources of infection or behavior leading to virus spread are warranted (17, 18). The utility of viral sequencing to track norovirus in transmission studies has been explored with fragments of the viral genome (19–22). As a consequence of the speed of disease onset and high transmissibility, the number of nucleotide and amino acid sequence changes within a local outbreak may be rare, so the sequencing of larger genomic fragments should provide greater resolution for defining transmission patterns.

The natural duration and specificity of immune responses to norovirus are difficult to measure because of the lack of a cell culture system for norovirus neutralization studies and the inability to grow a defined virus for such trials (reviewed in references 16 and 23). The duration of norovirus immunity may be limited by the short period of a typical infection and a correspondingly short exposure to viral antigens. Periodic population level replacement of norovirus lineages with viruses with surface residues under positive selection is evidence of immune response-driven antigenic change and suggests that these immune responses are of sufficient strength to drive viral evolution (24–26). Immune studies have identified blockade epitopes in VP1, the major capsid protein. These epitopes are important for interaction with HBGAs on target host cells; high titers of antibodies that block virus-like particle binding to HBGAs correlate with protection from a norovirus challenge (27–29).

Diarrheal diseases are a serious health problem, especially in developing countries when combined with nutritional problems, coinfection with other pathogens, crowding, and limited access to health care. It is clear that norovirus and rotavirus are frequently associated with diarrhea in this setting (30), and it is essential to closely follow the local evolution of norovirus. We describe here a method for deep sequencing of the approximately 7,500-nucleotide (nt) norovirus RNA genome directly from patient material and use this method to provide a detailed description of genome- and community-wide norovirus evolution.

## MATERIALS AND METHODS

### Primer design.

**FIG 1:**
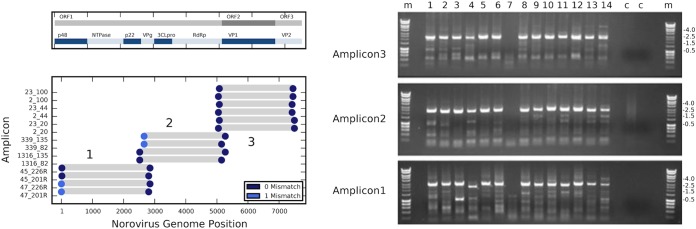
Primer design and function for full-genome deep-sequencing amplification. (Left panel) Virtual PCR showing the mapping of the designed primers to a norovirus GII.4 genome (GenBank accession no. JQ613552). Colored circles indicate the position of each primer and the number of mismatches; gray bars indicate the predicted sizes of the PCR products. A schematic of the ORF organization of the virus is shown at the top. (Right panel) The PCR products from 14 samples for individual primer pairs for amplicons 1, 2, and 3. Amplification of sample 7 failed. Lanes: c, water control; m, size marker. The sizes of relevant marker bands (in kilobase pairs) are indicated to the right.

Primers were designed by using Python algorithms to identify highly conserved primer targets in the appropriate genome locations. Briefly, the algorithm takes as input all of the complete human norovirus genome sequences available in the GenBank database (January 2012, 260 GII.4 entries, 5 GI entries; total sequence, 1.9 × 10^6^ nt). A counting method was employed to identify all of the highly conserved primer-like sequences with G+C percentages between 30 and 75%, calculated melting temperatures (*T_m_*s) between 55 and 59°C, and no single nucleotide comprising greater than 40% of the sequence. The norovirus genome was divided into three overlapping 2.5- to 3-kb amplicons, and the highest-frequency primer sites in the first and last 800 nt of each amplicon were selected. Finally the primers were used in a virtual PCR to determine the binding behavior of the primer set with all of the available full norovirus genomes (see Fig. 1). Primer details are summarized in Table 1.

**TABLE 1:** Primers used in this study

Primer	Sequence	Strand	Position^*a*^	*T_m_*^*b*^	GC_fraction	Norovirus GII genomes (517)^*c*^		Norovirus genomes (753)^*f*^	
						0 MM^*d*^	0–3 MM^*e*^	0 MM^*g*^	0–3 MM^*h*^
UNP_47	GTGAATGAAGATGGCGTCTAAC	Plus	1	55.52	0.45	98	100	83	84
UNP_45	TCTAACGACGCTTCCGCTG	Plus	17	58.30	0.58	75	96	62	80
UNP_201R	GCAATGGCCACCTCCTCAT	Minus	2808	57.95	0.58	97	100	80	85
UNP_226R	TTGGCCTCCTCCTCTTCACA	Minus	2850	58.21	0.55	92	99	76	82
UNP_339	GGCAAGAAGCACACAGCC	Plus	2660	57.48	0.61	88	92	73	78
UNP_1316	TGGTCCAAGCCACAAGTGG	Plus	2519	58.05	0.58	11	100	13	89
UNP_82	GACCTCTGGGACGAGGTTG	Minus	5150	57.41	0.63	87	96	70	80
UNP_135	CTCCACCAGGGGCTTGTAC	Minus	5271	57.63	0.63	89	94	73	78
UNP_2	GGGAGGGCGATCGCAAT	Plus	5049	57.57	0.65	88	96	72	79
UNP_23	TTGTGAATGAAGATGGCGTCGA	Plus	5079	58.53	0.45	56	100	42	84
UNP_100	GCCAGTCCAGGAGTCCAA	Minus	7447	56.43	0.61	74	97	61	83
UNP_44	GCACGGTTGAGACTGTGC	Minus	7418	57.27	0.61	84	98	69	82
UNP_20	CGAGGGGAGTCACGGGT	Minus	7493	58.34	0.71	86	97	70	83

a Primer mapping position in the norovirus GII.4 genome (GenBank accession no. JQ613552).

b The *T_m_* was calculated with a Python script that approximates the Breslauer method (59).

c All GenBank database entries (July 2014) for norovirus GII (taxonomic identification no. 142786; length, 7,000 to 8,000 nt; 517 entries).

d Percentage of norovirus GII genomes (*n* = 517) showing perfect homology to the primer.

e Percentage of norovirus GII genomes (*n* = 517) showing the target sequence for the primer with up to three mismatches.

f All GenBank database entries (July 2014) for norovirus (taxonomic identification no. 122929; length, 7,000 to 8,000 nt; 753 entries).

g Percentage of norovirus genomes (*n* = 753) showing perfect homology to the primer.

h Percentage of norovirus genomes (*n* = 753) showing the target sequence for the primer with up to three mismatches.

### Sample collection.

Stool samples were obtained as part of a larger study examining causes of pediatric diarrhea in subjects presenting to Children's Hospital 1, Children's Hospital 2, and the Hospital for Tropical Diseases, Ho Chi Minh City (HCMC), Vietnam (30, 31). Additional samples came from an *ad hoc* enrollment of children admitted to Children's Hospital 2 with potentially hospital-acquired norovirus diarrhea or prolonged norovirus incubation. In the *ad hoc* collection, pediatric patients were admitted to the hospital because of diseases other than diarrheal diseases and had no diarrhea when they arrived at the hospital. The group included only patients who developed diarrhea after at least 48 h of hospitalization, with the diarrhea lasting at least 3 days after onset. Ethical approval was granted by the institutional ethical review boards and the University of Oxford Tropical Research Ethics Committee (OxTREC no. 0109).

### Generation of amplified cDNA for deep sequencing.

For RNA extraction, 140 μl of each stool specimen was subjected to automated extraction into a final 50-μl elution with the MagNA Pure 96 automated extraction machine according to the manufacturer's instructions (Roche). Reverse transcription (RT) was performed as previously described (32). Briefly, a primer mixture was prepared separately for each amplicon; the reverse primers for the amplicon were pooled in an equimolar ratio and water added up to 7 μl of the primer mixture (7.6 pmol of each primer; 0.38 pmol/μl per reaction mixture). Extracted norovirus RNA was diluted 1:10 in water; 5 μl of this dilution was added to the primer mixture, which was heated for 5 min at 65°C and immediately transferred to an ice block for 1 min. An enzyme mixture was then added to each reaction mixture and mixed by pipetting. Each 20-μl reaction mixture contained 4 μl of 5× First Strand buffer (250 mM Tris-HCl [pH 8.3], 375 mM KCl, 15 mM MgCl_2_), 1 μl of 0.1 M dithiothreitol, 1 μl of 10 mM deoxynucleoside triphosphates, 1 μl of RNase Inhibitor (40 U/μl; Promega), and 1 μl of SuperScript III reverse transcriptase (200 U/μl; Life Technologies). RT was performed at 50°C for 60 min, followed by 70°C for 15 min.

### PCR amplification.

Amplification was performed with primer mixture solutions prepared for each amplicon. For the primer mixture (per 25-μl reaction mixture), the forward and reverse primers from each amplicon were pooled in a 1.5:1 ratio (1.9 pmol of each forward primer and 1.26 pmol of each reverse primer; 0.08 pmol/μl and 0.05 pmol/μl, respectively). A 5-μl aliquot of the RT reaction mixture for each amplicon was used as the template for the PCR step. The thermal cycling conditions used were enzyme activation at 98°C for 30 s; 35 cycles of 98°C for 10 s, 53°C for 30 s, and 72°C for 3.0 min; a final extension at 72°C for 10 min; and holding at 4°C.

### Sequencing and genome assembly.

Pooled amplicons for each sample (approximately 1.2 μg) were individually indexed and subjected to sequencing with Illumina MiSeq (33, 34) to generate approximately 300,000 reads of 149 nt per sample (median value, 302,904 reads). All reads were processed with QUASR (35) to remove sequencing adapters and index sequences and to trim primer sequences present within a fixed distance of the 5′ or 3′ end of a read. Reads were then trimmed from the 3′ end to reach a minimum median Phred quality score of 35, and reads <125 nt in length were removed. After primer trimming and quality control for each sample, *de novo* assembly with SPAdes (36) was used to generate full norovirus genomes. Intact ORFs were checked with Python scripts as a measure of correct genome assembly.

### Recombination detection.

The 119 complete genomes of all of the GII noroviruses from this study and from global data (retrieved from the GenBank database) were manually aligned with Se-AL v2.0 (http://tree.bio.ed.ac.uk/software/seal/). Only full-length sequences with information on the sample collection date and location were included in this analysis. The potential presence of recombination in these complete sequences was screened for with the Recombination Detection Program version 4 (RDP4) software (37). The RDP, GENECONV, 3SEQ, and MAXCHI methods were employed for primary screening, and the BOOTSCAN and SISCAN methods were used for automatic checking of the recombination signals, as described previously (38). The automask X function in RDP4 was selected for optimal recombination detection; i.e., one representative strain within each group of similar sequences was examined during the primary/exploratory search for recombination signals while the remaining sequences within groups of sequences with high similarity were automatically masked. By this method, masked sequences were examined for the presence of recombination if the program detected a recombination signal in the representative unmasked sequence. Each test of recombination used a 400-nt sliding window, and any recombination signals with significant *P* values for three or more test parameters were considered potential recombination events. A further analysis of these potential recombinants, comparing tree topologies with likelihood (Shimodaira-Hasegawa test) was employed to determine which of the test strains were likely to be true recombinants and which were not. All intra-ORF recombinant strains (GenBank accession numbers EU921388, AB541275, GU991355, and AB541254) were excluded from the estimation of positive selection and evolutionary rates.

### Phylogenetic analysis.

An alignment of nonrecombinant sequences including all of the full genomes determined in this analysis and global background sequences obtained from the GenBank database was utilized to reconstruct evolutionary relationships among norovirus sequences. A phylogenetic tree was inferred by using aligned nucleotide sequences, employing a maximum-likelihood (ML) method in RaxML (39) under the GTR+Γ model of substitution, which was determined to be the model that fit our data best with jModelTest version 2.1.1 (40). Tree topology was assessed through bootstrapping with 1,000 pseudoreplicates. The resulting phylogenetic tree was visualized and edited in FigTree v1.4.0 (http://tree.bio.ed.ac.uk/software/figtree/).

### Evolutionary-rate estimations.

Evolutionary rates were estimated by a Bayesian Markov chain Monte Carlo (BMCMC) method implemented in BEAST version 1.7.2 (41). A relaxed uncorrelated lognormal molecular clock was employed to account for lineage-specific rates, and a GMRF Bayesian skyride coalescent (42) was used to model the population dynamics. The relevant substitution models for each alignment were selected with jModelTest version 2.1.1 (40). The mean evolutionary rate and the 95% upper and lower highest posterior density (HPD) intervals were inferred from the posterior tree distribution generated from the BMCMC runs with Tracer version 1.6 (http://tree.bio.ed.ac.uk/software/tracer/).

### Positive-selection analysis.

To determine evolutionary patterns of norovirus, selection analyses of the regions encoding VP1, VP2, and the ORF1-encoded p48 (NS1-2) and p22 (NS4) proteins were performed. Norovirus codons under selective pressure were first determined with the mixed-effects model of evolution (MEME; *P* value, <0.05) (43) and fast unconstrained Bayesian approximation (FUBAR; posterior probability, >0.9) (44) implemented through the DataMonkey web browser (45). Codons that were found to be under positive selection by either method were inspected at the sequence alignment, and those with no evidence of polymorphisms were considered false positive and discarded.

Ancestral sequences were reconstructed from the sequence alignment and inferred phylogeny by the joint-likelihood method implemented in HyPhy (46) under a GTR+Γ model of evolution.

### Nucleotide sequence accession numbers.

**FIG 2:**
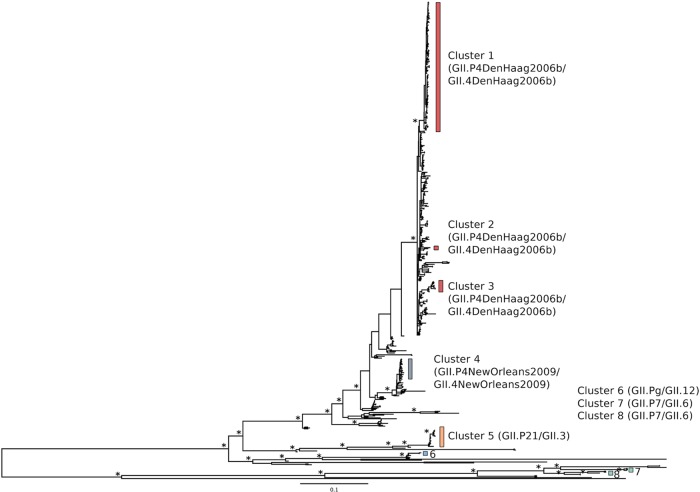
ML phylogenetic tree of the 112 HCMC GII genomes in this study and 89 GII.4 genomes from the same HCMC cohort that were sequenced separately and made publically available in the GenBank database plus selected global reference genomes. The eight phylogenetic clusters of norovirus identified in this study are marked with colored bars. Bootstrap support of ≥0.85 at key nodes is indicated with asterisks. The tree is midpoint rooted for clarity, and all horizontal branch lengths are drawn to a scale of nucleotide substitutions per site.

The GenBank accession numbers of all of the new norovirus sequences reported here are listed in Table 2. Also listed are the sample collection dates, the genetic clusters (see Fig. 2 and Table 4), and the European Nucleotide Archive accession numbers of the raw sequence data. In addition, 89 GII.4 genomes from the same HCMC study are publically available in the GenBank database, with the following accession numbers: cluster 1, KC409244, KC409245, KC409246,KC409257, KC409258, KC409259, KC409260, KC409261, KC409262, KC409264, KC409265, KC409266, KC409267, KC409268, KC409269, KC409270, KC409271, KC409272, KC409273, KC409274, KC409275, KC409276, KC409277, KC409279, KC409280, KC409281, KC409282, KC409283, KC409284, KC409285, KC409286, KC409287, KC409288, KC409289, KC409290, KC409291, KC409293, KC409294, KC409295, KC409296, KC409297, KC409298, KC409304, KC409305, KC409306, KC409307, KC409308, KC409309, KC409310, KC409312, KC409313, KC409314, KC409315, KC409318, KC175360, KC175365, KC175366, KC175371, KC175373, KC175381, KC175388, KC175389, KC175390, KC175391, KC175392, KC175393, KC175394, KC175395, KC175396, KC175406, KC175407, KC175408, KC175409, and KC175410; cluster 3, KC409256, KC409263, and KC409278; cluster 4, KC409240, KC409241, KC409242, KC409243, KC409299, KC409301, KC409302, KC409303, KC175384, KC175385, KC175386, and KC175387.

**TABLE 2:** GenBank and ENA accession numbers, genetic cluster, and sample collection date

Virus	GenBank accession no.^*a*^	ENA accession no.^*b*^	Cluster^*c*^	Sample collection date^*d*^
Hu_GII_10116_2009_VNM	KM198480	ERR212491	1	9/7/2009
Hu_GII_10054_2009_VNM	KM198481	ERR225641	1	21/5/2009
Hu_GII_10114_2009_VNM	KM198482	ERR212490	1	9/7/2009
Hu_GII_10313_2010_VNM	KM198483	ERR217285	4	22/2/2010
Hu_GII_30212_2009_VNM	KM198484	ERR223539	5	6/10/2009
Hu_GII_10148_2009_VNM	KM198485	ERR212498	2	11/8/2009
Hu_GII_C2H-18_2011_VNM	KM198486	ERR225628	3	30/8/2011
Hu_GII_10110_2009_VNM	KM198487	ERR212489	1	6/7/2009
Hu_GII_10325_2010_VNM	KM198488	ERR217290	4	26/2/2010
Hu_GII_10101_2009_VNM	KM198489	ERR212487	1	25/6/2009
Hu_GII_10002_2009_VNM	KM198490	ERR225635	1	4/5/2009
Hu_GII_30351_2009_VNM	KM198491	ERR138007	4	17/12/2009
Hu_GII_30448_2010_VNM	KM198492	ERR223547	6	29/1/2010
Hu_GII_30468_2010_VNM	KM198493	ERR223549	5	24/2/2010
Hu_GII_10247_2009_VNM	KM198494	ERR217278	1	10/12/2009
Hu_GII_10193_2009_VNM	KM198495	ERR138002	1	05/10/2009
Hu_GII_20419_2010_VNM	KM198496	ERR223554	5	1/2/2010
Hu_GII_10236_2009_VNM	KM198497	ERR217280	1	19/11/2009
Hu_GII_20088_2009_VNM	KM198498	ERR223553	8	28/7/2009
Hu_GII_20118_2009_VNM	KM198499	ERR212481	1	28/8/2009
Hu_GII_C2H-20_2011_VNM	KM198500	ERR225629	5	5/9/2011
Hu_GII_10173_2009_VNM	KM198501	ERR212503	1	11/9/2009
Hu_GII_10136_2009_VNM	KM198502	ERR212495	1	3/8/2009
Hu_GII_C2033_2010_VNM	KM198503	ERR212484	6	28/6/2010
Hu_GII_20151_2009_VNM	KM198504	ERR212467	1	16/9/2009
Hu_GII_20460_2010_VNM	KM198505	ERR223530	5	4/3/2010
Hu_GII_10199_2009_VNM	KM198506	ERR217283	1	20/10/2009
Hu_GII_C2007_2010_VNM	KM198507	ERR138011	4	2/4/2010
Hu_GII_20066_2009_VNM	KM198508	ERR212470	1	14/7/2009
Hu_GII_20479_2010_VNM	KM198509	ERR223531	5	16/3/2010
Hu_GII_10012_2009_VNM	KM198510	ERR225637	1	7/5/2009
Hu_GII_C2H-24_2011_VNM	KM198511	ERR225631	5	16/9/2011
Hu_GII_10062_2009_VNM	KM198512	ERR225642	1	29/5/2009
Hu_GII_20494_2010_VNM	KM198513	ERR223534	1	19/3/2010
Hu_GII_10079_2009_VNM	KM198514	ERR212486	1	11/6/2009
Hu_GII_C2H-31_2011_VNM	KM198515	ERR217269	3	28/9/2011
Hu_GII_10285_2010_VNM	KM198516	ERR217288	4	18/1/2010
Hu_GII_30399_2010_VNM	KM198517	ERR138008	1	11/1/2010
Hu_GII_10182_2009_VNM	KM198518	ERR212507	1	17/9/2009
Hu_GII_30082_2009_VNM	KM198519	ERR223537	8	23/6/2009
Hu_GII_10158_2009_VNM	KM198520	ERR212499	1	21/8/2009
Hu_GII_10176_2009_VNM	KM198521	ERR212504	1	14/9/2009
Hu_GII_20150_2009_VNM	KM198522	ERR212466	1	15/9/2009
Hu_GII_10204_2009_VNM	KM198523	ERR217287	1	29/10/2009
Hu_GII_10034_2009_VNM	KM198524	ERR225638	1	15/5/2009
Hu_GII_10163_2009_VNM	KM198525	ERR212501	1	28/8/2009
Hu_GII_10075_2009_VNM	KM198526	ERR225644	1	9/6/2009
Hu_GII_10074_2009_VNM	KM198527	ERR225643	1	9/6/2009
Hu_GII_C2H-25_2011_VNM	KM198528	ERR225632	5	20/9/2011
Hu_GII_C2H-27_2011_VNM	KM198529	ERR225633	5	21/9/2011
Hu_GII_20486_2010_VNM	KM198530	ERR223532	7	18/3/2010
Hu_GII_30116_2009_VNM	KM198531	ERR223538	7	9/7/2009
Hu_GII_10108_2009_VNM	KM198532	ERR212488	1	2/7/2009
Hu_GII_30241_2009_VNM	KM198533	ERR223540	1	26/10/2009
Hu_GII_30443_2010_VNM	KM198534	ERR223546	8	28/1/2010
Hu_GII_20092_2009_VNM	KM198535	ERR212474	1	31/7/2009
Hu_GII_20079_2009_VNM	KM198536	ERR212473	1	24/7/2009
Hu_GII_10137_2009_VNM	KM198537	ERR212496	1	4/8/2009
Hu_GII_10051_2009_VNM	KM198538	ERR225640	1	21/5/2009
Hu_GII_C2H-36_2011_VNM	KM198539	ERR217266	3	25/10/2011
Hu_GII_20145_2009_VNM	KM198540	ERR212464	1	11/9/2009
Hu_GII_20188_2009_VNM	KM198541	ERR138004	1	7/10/2009
Hu_GII_20094_2009_VNM	KM198542	ERR212476	2	3/8/2009
Hu_GII_20357_2009_VNM	KM198543	ERR138005	4	30/12/2009
Hu_GII_C2418_2010_VNM	KM198544	ERR138012	4	1/11/2010
Hu_GII_10195_2009_VNM	KM198545	ERR217284	1	13/10/2009
Hu_GII_20067_2009_VNM	KM198546	ERR212471	1	15/7/2009
Hu_GII_C2H-47_2011_VNM	KM198547	ERR217275	5	3/11/2011
Hu_GII_20107_2009_VNM	KM198548	ERR212477	1	20/8/2009
Hu_GII_30473_2010_VNM	KM198549	ERR223550	7	1/3/2010
Hu_GII_10078_2009_VNM	KM198550	ERR225645	1	10/6/2009
Hu_GII_20108_2009_VNM	KM198551	ERR212478	1	20/8/2009
Hu_GII_20154_2009_VNM	KM198552	ERR212469	1	16/9/2009
Hu_GII_30381_2010_VNM	KM198553	ERR223544	5	4/1/2010
Hu_GII_C2H-48_2011_VNM	KM198554	ERR217271	5	4/11/2011
Hu_GII_C2035_2010_VNM	KM198555	ERR138006	4	28/6/2010
Hu_GII_10127_2009_VNM	KM198556	ERR212492	1	17/7/2009
Hu_GII_10194_2009_VNM	KM198557	ERR217286	1	13/10/2009
Hu_GII_30257_2009_VNM	KM198558	ERR223541	1	30/10/2009
Hu_GII_10129_2009_VNM	KM198559	ERR212493	1	20/7/2009
Hu_GII_C2H-50_2011_VNM	KM198560	ERR217268	3	22/11/2011
Hu_GII_30303_2009_VNM	KM198561	ERR223542	5	23/11/2009
Hu_GII_C2H-55_2011_VNM	KM198562	ERR212509	3	25/11/2011
Hu_GII_C2365_2010_VNM	KM198563	ERR212485	5	15/9/2010
Hu_GII_C2H-44_2011_VNM	KM198564	ERR217276	3	31/10/2011
Hu_GII_10169_2009_VNM	KM198565	ERR212502	1	4/9/2009
Hu_GII_20093_2009_VNM	KM198566	ERR212475	1	3/8/2009
Hu_GII_10255_2009_VNM	KM198567	ERR217273	1	15/12/2009
Hu_GII_C2H-62_2011_VNM	KM198568	ERR217267	3	14/12/2011
Hu_GII_10235_2009_VNM	KM198569	ERR217274	1	19/11/2009
Hu_GII_20146_2009_VNM	KM198570	ERR212465	1	11/9/2009
Hu_GII_20123_2009_VNM	KM198571	ERR212479	1	1/9/2009
Hu_GII_20370_2010_VNM	KM198572	ERR212461	5	6/1/2010
Hu_GII_C2H-45_2011_VNM	KM198573	ERR217277	5	2/11/2011
Hu_GII_10183_2009_VNM	KM198574	ERR217289	1	22/9/2009
Hu_GII_20069_2009_VNM	KM198575	ERR212472	1	16/7/2009
Hu_GII_C2H-43_2011_VNM	KM198576	ERR217281	3	14/10/2011
Hu_GII_10145_2009_VNM	KM198577	ERR212497	1	7/8/2009
Hu_GII_C2H-52_2011_VNM	KM198578	ERR212508	3	24/11/2011
Hu_GII_10160_2009_VNM	KM198579	ERR212500	1	26/8/2009
Hu_GII_10223_2009_VNM	KM198580	ERR217272	1	6/11/2009
Hu_GII_10003_2009_VNM	KM198581	ERR225636	1	5/5/2009
Hu_GII_30192_2010_VNM	KM198582	ERR138003	1	21/9/2009
Hu_GII_20493_2010_VNM	KM198583	ERR223533	5	19/3/2010
Hu_GII_10131_2009_VNM	KM198584	ERR212494	1	22/7/2009
Hu_GII_10238_2009_VNM	KM198585	ERR217282	1	19/11/2009
Hu_GII_30400_2010_VNM	KM198586	ERR223545	5	11/1/2010
Hu_GII_20153_2009_VNM	KM198587	ERR212468	1	16/9/2009
Hu_GII_10037_2009_VNM	KM198588	ERR225639	1	15/5/2009
Hu_GII_20144_2009_VNM	KM198589	ERR212463	1	10/9/2009
Hu_GII_C2H-39_2011_VNM	KM198590	ERR217270	5	24/10/2011
Hu_GII_20122_2009_VNM	KM198591	ERR212480	1	31/8/2009

a GenBank accession number, accessible at http://www.ncbi.nlm.nih.gov/nuccore/.

b European Nucleotide Archive (ENA) accession number, accessible at http://www.ebi.ac.uk/ena/.

c Genetic cluster as defined in Fig. 2.

d Day/month/year of sample collection.

## RESULTS

### Norovirus sequencing strategy.

A novel general strategy for designing PCR primers was developed that would permit the production of complete norovirus genome sequences. Deep sequencing of RNA virus genomes requires RT of viral RNA and amplification of the resulting cDNA, which encompasses the entire viral genome. Python algorithms were used to process all of the available norovirus full-genome data (265 full genomes, January 2012) and to select primer target sequences suitable for whole-genome amplification. Briefly the algorithm processes the norovirus sequence data into primer-sized sequences trimmed to a calculated *T_m_*. The frequency of each sequence in the entire set is calculated, with high-frequency sequences correlating with conserved sites across the viral genome. The norovirus genome was divided into three overlapping amplicons, potential primers were mapped to a reference genome, and the highest-frequency sequences mapping within the terminal 800 nt of each amplicon were identified. Reverse complements of the primers mapping to the 3′ end were prepared. A virtual PCR was performed to examine the potential function of the primers across all known full norovirus genomes. The output of such an analysis is shown in the left panel of Fig. 1, with blue markers indicating the position of each primer and gray bars indicating the expected PCR product. The actual function of the primer set is demonstrated in the right panel of Fig. 1, with each lane showing the PCR products from 14 samples, present by amplicon. Each RT reaction mixture contained two (or three for amplicon 3) reverse primers each for amplicon 1, 2, or 3, and each PCR mixture contained two (or three for amplicon 3) forward and reverse primers for amplicon 1, 2, or 3. Of these samples, no. 7 failed; however, the remaining 13 samples provided sufficient material for deep sequencing.

A summary of the predicted performance of the norovirus primer set with all of the available norovirus genomes is shown in Table 1. All full-length norovirus GII genomes (taxonomic identification no. 142786; length, 7,000 to 8,000 nt; 517 entries) or all norovirus genomes (taxonomic identification no. 122929; length 7,000 to 8,000 nt; 753 entries) were retrieved from the GenBank database. These genome sets were examined for the target sequence for each primer, and the percentage of genomes with a perfect match to the target sequence or with a functional match (zero to three mismatches) to the target sequence was reported. For the norovirus GII genomes, the primers have a perfect match to 79% of the genomes and a functional match (up to 3 mismatches) to 97% of the genomes. For the complete set of norovirus genomes (this includes all GI, all GII, and all animal noroviruses), the primers have a perfect match to 65% of the genomes and a functional match (zero to three mismatches) to 82% of the genomes. These values and the details of the analysis, as well as the GC contents and calculated *T_m_*s for all of the primers, are listed in Table 1.

A summary of the performance of the norovirus primer set for amplifying and sequencing 188 fecal sample-derived RNAs is presented in Table 3. PCR success was defined as obtaining the three amplicon-specific RT-PCR products of the predicted size with sufficient yield for sequencing library preparation. The overall RT-PCR success rate was 78.2% (147 of the 188 clinical samples tested). The most common genotype globally, GII.4, had the highest PCR success rate (93.7%, 74 of 79 samples), followed by GII.6 (88%, 7 of 8 samples), GII.13 (83%, 5 of 6 samples), and GII.3 (77%, 26 of 34 samples). Much lower amplification efficiency was observed for GI strains, with successful PCR genome amplification in only 2 of 10 samples tested. The high success with GII with respect to GI strains (especially GII.4) was predicable given that GII.4 genomes dominate the sequences in public databases. Future primer sets could be reiteratively designed by using targeted and revised genome data sets.

**TABLE 3:** PCR and genome sequencing success by norovirus genotype

Genotype^*a*^	No. of samples	Amplicon 1^*b*^	Amplicon 2^*b*^	Amplicon 3^*b*^	No. of genomes^*c*^	(%) Successful
GII.4	60	55	55	57	55	92
GII (non-GII.4)	58	48	45	53	45	74
GI	10	7	4	4	2	20
GII.2	5	4	1	5	2	40
GII.3	34	26	27	27	26	77
GII.6	8	8	7	8	7	88
GII.7	2	2	0	2	0	0
GII.9	1	1	0	1	1	100
GII.12	2	1	2	2	1	50
GII.13	6	5	6	6	5	83

a Sample genotype previously determined (30, 31).

b Successful RT-PCR amplification of sufficient DNA (ca. 0.4 μg) for Illumina library preparation. The values shown are the number of successful amplicons generated.

c Yield of >95% of the full genome.

### Norovirus diversity in HCMC.

By using the whole-genome sequencing technique developed, 112 novel GII norovirus genomic sequences were generated. In addition, 89 GII.4 genomes from the same HCMC study were also publically available in the GenBank database; these were included in the following analysis for a total of 201 complete genomes with collection dates between April 2009 and December 2011. A phylogenetic analysis of the 201 genomes defined eight genotypes of GII norovirus by ML methods (Fig. 2). Consistent with previous characterization of norovirus infections in HCMC (30) and global norovirus patterns, the most prevalent GII.4 genotype found in this study belonged to the GII.4 Den Haag lineage (Fig. 2, clusters 1, 2, and 3), which is most genetically similar to the GII.4 Minerva_2006b partial sequence and Taiwan_2006 (GenBank accession no. JN400601). Phylogenetically, the GII.4 strains in cluster 4 (Fig. 2A) were most closely related to GII.4 New_Orleans_2010 (GenBank accession no. JN595867), while the GII strains in cluster 5 were classified as GII.P21_GII.3 and most closely related to strain NV_Pune_2007 (GenBank accession no. EU921389). A small number of strains belonged to genotype GII.Pg_GII.12 (cluster 6), while viruses of the GII.P7_GII.6 genotype fell into two distinct lineages, clusters 7 and 8 (Fig. 2). Our genotype assignment based on phylogenetic reconstruction was consistent with the genotype designation generated by the RIVM algorithm (47) (Table 4). Additionally, the relative frequency of each genotype observed in the full genome set was similar to the frequencies determined by My et al. (31) from a larger set of HCMC samples with ORF1 and -2 fragments (Table 4), indicating that the generation of full genome sequences was not strongly influenced by genotype-based selection biases. Viruses of the GII.4 Den Haag and GII.P21/GII.3 genotypes (clusters 1 and 5) were identified in two sampling periods, in 2009-2010 and later in 2011, while the other virus genotypes were detected only in the first sampling period.

**TABLE 4:** Phylogenetic clusters identified in this study

Phylogenetic cluster^*a*^	Closest genome^*b*^	Genotype by RIVM algorithm^*c*^	No. of genomes	Frequency (%) in 201 genomes
1	NV_GII_VNM_2009_KC175360	GII.P4.DH06b_GII.4.DH06b	140	69.65 (67.6)^*d*^
	NV_GII_VNM_2009_KC175395			
2	NV_GII_VNM_2009_KC175402	GII.P4.DH06b_GII.4.DH06b	2	
3	NV_GII4_TW_2007_JN400615	GII.P4.DH06b_GII.4.DH06b	12	
	NV_GII4_Ehime_2007_AB541241			
4	NV_GII_VNM_2010_KC175383	GII.P4.NO09_GII.4.NO09	20	9.95 (9.5)
5	NV_Pune_2007_EU921389	GII.P21_GII.3	19	9.45 (10.2)
6	NV_Pune_2007_EU921389	GII.Pg_GII.12	2	0.99 (0.6)
	NV_GII2_12_Wahroonga_2009_JQ613568			
7	NV_GII_Gifu_1999_AB084071 (<50%)	GII.P7_GII.6	3	1.49 (2.5)
8	NV_GII_Gifu_1999_AB084071 (<50%)	GII.P7_GII.6	3	1.49 (2.5)

a Phylogenetic classification (see Fig. 2).

b Based on the number of reads mapped.

c Based on the RIVM algorithm (47).

d The values in parentheses are genotype frequency percentages determined by My et al. (31).

**FIG 3:**
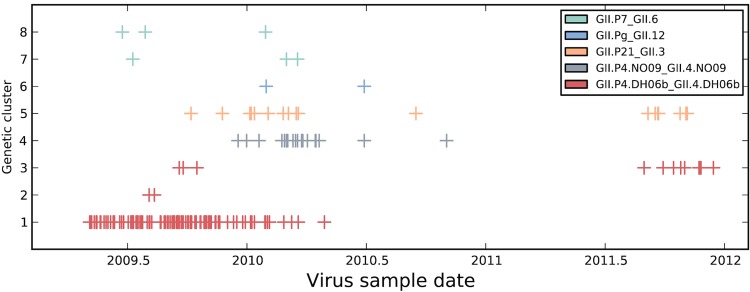
Temporal appearance of the HCMC norovirus GII genotypes during the study period. Genomes were stratified by genotype (from Fig. 2), color coded, and plotted by date of sample isolation.

The temporal occurrence of sampled noroviruses is shown in Fig. 3, with samples stratified by genotype cluster. The three GII.P4/GII.4(2006) genotypes (clusters 1, 2, and 3) were present in the first half of 2009, with the GII.P4/GII.4(2010) genotype (cluster 4, gray) first appearing at the end of 2009. There was a pause in sampling in the first half of 2011, followed by sampling in the second half of 2011. Reduced diversity was observed in 2011, with only clusters 3 and 5 sequenced from these samples. Changes in sampling protocols between 2010 and 2011 preclude inference of how this reduced diversity may relate to norovirus epidemiology and evolution. However, the identification of clusters of phylogenetically related viruses undergoing *in situ* evolution in this region over the observation period allowed an examination of evolutionary processes that may allow the continued transmission and maintenance of viral lineages in the presence of population immune responses. Characterization of such changes in the norovirus population may provide important clues about how the virus evades host immunity.

### Evolutionary rates within each cluster.

**FIG 4:**
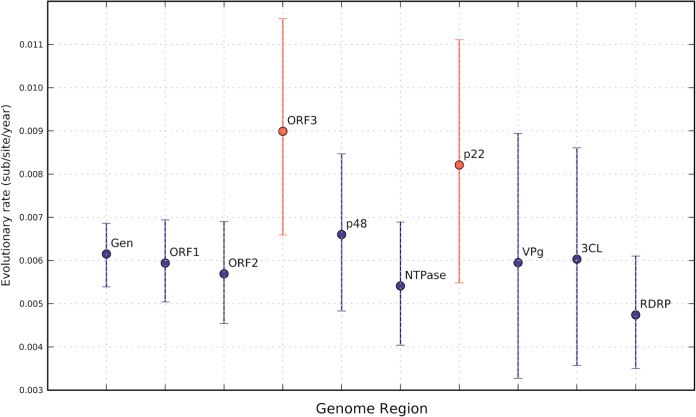
Summary of evolutionary rates inferred for the genomic regions of GII.4 cluster 1. Evolutionary rates were estimated as described in Materials and Methods, and mean values are indicated by colored circles, and error bars show 95% confidence intervals. The region of the norovirus genome used for calculation is labeled, and the two regions with rates higher than that of the full genome are in red.

A sufficient number of genomes were available from clusters 1, 4, and 5 for well-supported evolutionary-rate estimations (Table 5). Mean evolutionary rates of 6.15 × 10^−3^, 5.73 × 10^−3^, and 5.34 × 10^−3^ substitution per site per year were estimated from the full genomes of clusters 1, 4, and 5. Figure 4 plots the rates for the GII.4 cluster 1 viruses by the region of the genome used for each calculation.

**TABLE 5:** Evolutionary rates

Sequence set and genomic region	Mean rate (95% HPD)^*a*^	Substitution model
Cluster 1, GII.P4 Den Haag 2006b_GII.4 Den Haag 2006b		
Whole genome	6.15 (5.39–6.86)	SRD06
ORF1	5.94 (5.04–6.94)	SRD06
ORF2	5.69 (4.54–6.90)	SRD06
ORF3	8.99 (6.59–11.6)	SRD06
p48 (NS1-2)	6.60 (4.83–8.47)	GTR+G
NTPase (NS3)	5.41 (4.04–6.89)	GTR+G
p22 (NS4)	8.21 (5.48–11.11)	GTR+G
VPg (NS5)	5.95 (3.27–8.94)	HKY+G
3CLpro (NS6)	6.03 (3.57–8.61)	GTR+G
RdRp (NS7)	4.74 (3.50–6.10)	GTR+G
Cluster 4, GII.P4 New Orleans 2009_GII.4 New Orleans 2009		
Whole genome	5.73 (3.74–7.81)	GTR+G
ORF1	4.03 (1.77–6.33)	HKY+G
ORF2	5.60 (0.68–9.82)	HKY+G
ORF3^*b*^		
Cluster 5, GII.P21_GII.3		
Whole genome	5.34 (4.06–6.82)	SRD06
ORF1	4.81 (3.45–6.17)	SRD06
ORF2	5.99 (3.75–8.39)	SRD06
ORF3	7.38 (2.06–13.9)	SRD06

a Evolutionary rates were measured as 10^−3^ substitution per site per year. The mean evolutionary rate (10^−3^ substitution per site per year) and the 95% upper and lower HPD intervals were determined as described in Materials and Methods.

b There was insufficient signal for the algorithms to return a reliable evolutionary rate for ORF3 region of sequences from GII.4 cluster 4.

The ORF-specific rates estimated for the three genetic clusters show that the ORF1 regions exhibited a lower rate than those of the ORF2 (VP1) regions. For all three clusters, the ORF1 and ORF2 (VP1) regions showed rates modestly lower than that of the full genome, while the ORF3 (VP2) substitution rates of both cluster 1 (8.99 × 10^−3^ substitution per site per year) and cluster 5 viruses (7.38 × 10^−3^ substitution per site per year) were higher than that of the whole genome. The overlapping confidence intervals for these estimations make these conclusions less secure. The amount of signal available for cluster 4 ORF3 was not sufficient to yield a reliable rate estimate.

Norovirus ORF1 encodes a large polyprotein containing the viral polymerase and protease and several essential replicase components. Evolutionary rates were estimated separately for these individual coding regions of cluster 1 ORF1 (Table 5; Fig. 4). The region encoding p22 (NS4) showed the highest levels of change (6.60 × 10^−3^ and 8.21 × 10^−3^ substitution per site per year, Fig. 4), greater than the whole-genome rates for cluster 1 (6.15 × 10^−3^ substitution per site per year). The enzymes (NTPase [NS3], protease, and RdRp [NS7]) and VP1 show substitution rates modestly lower than those observed across the whole genome.

### Amino acid changes in norovirus proteins.

The evolutionary patterns of four norovirus-encoded proteins with the higher evolutionary rates were examined (VP1, VP2, p48 [NS1-2], and p22 [NS4]). An alignment of protein sequences ordered by time was used to detect sustained versus sporadic changes in the protein relative to a reconstructed ancestral sequence. Information about the biochemical properties of the protein was gathered from the published literature. Positive-selection analysis was performed with MEME (43) or FUBAR (44).

**FIG 5:**
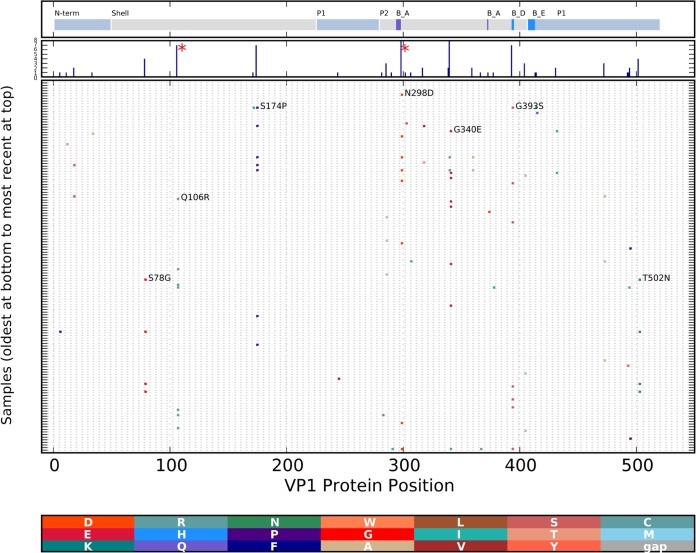
Changes in the GII.4 cluster 1 VP1 protein. The protein sequences were aligned, and amino acid differences from the reconstructed ancestral sequence of cluster 1 were determined and marked with vertical colored bars, with the new amino acid residue color coded as shown at the bottom; the gray bar indicates a gap in the query sequence. The sequences were ordered by sample date, with the earliest samples at the bottom of the graph. Functional domains of the VP1 protein are indicated at the top of the graph and include the shell domain and the protruding 1 (P1) and protruding 2 (P2) domains. The locations of blockade epitopes A and E are also indicated (B_A and B_E, respectively). The histogram in the second panel from the top indicates the total number of changes at each position. The protein changes occurring in more than four samples are annotated with the parental amino acid, the position, and the new amino acid. Codons found to be under positive selection by MEME or FUBAR are indicated with red asterisks.

Cluster 1 VP1 showed changes in multiple patients relative to the ancestral sequence, i.e., Q106R, S174P, and N298D in blockade epitope A and G340E and G393S in blockade epitope D (Fig. 5). Additional substitutions were seen at a lower frequency, suggesting evolution during the course of transmission through HCMC. Position 298 in blockade epitope A was found to be positively selected with FUBAR, while both FUBAR and MEME identified position 106 within the shell domain (Fig. 5) as being under positive selection (Table 6).

**TABLE 6:** Positive-selection analysis

Codon position^*a*^	FUBAR^*b*^	MEME^*c*^
ORF1 (p48 [NS1-2]) 79	0.991	0.037
ORF2 (VP1) 106	0.993	0.014
ORF2 (VP1) 298	0.984	>0.05
ORF3 (VP2) 144^*d*^	0.983	0.043

a Codons under positive selection in 140 cluster 1 norovirus genomes sequenced in this study as detected by FUBAR (44) and the MEME (43).

b Posterior probability values obtained by FUBAR are shown.

c *P* values obtained by MEME are shown.

d The analysis of ORF3 covered the first 247 of the protein's 268 codons.

**FIG 6:**
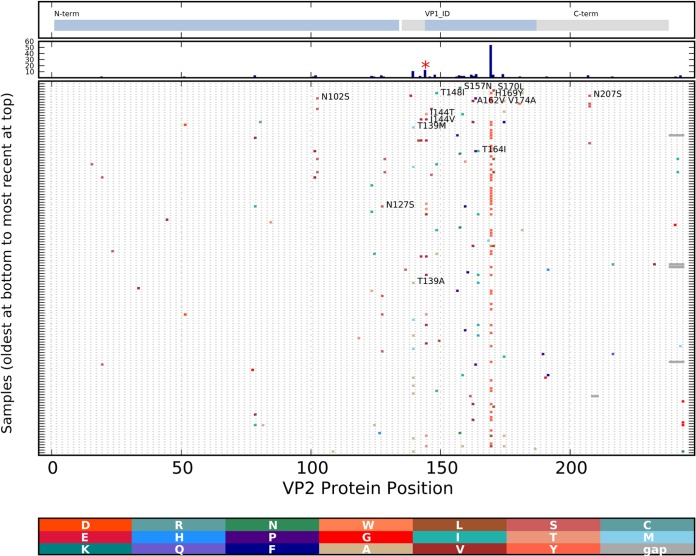
Changes in cluster 1 minor capsid protein VP2 over time. Protein changes were analyzed and are depicted as described in the legend to Fig. 5. The functional domains of the VP2 protein, including the VP1-interacting region (VP1_ID), are marked at the top. The histogram in the second panel from the top indicates the total number of changes at each position. A codon found to be under positive selection by the MEME or FUBAR is indicated with a red asterisk.

An alignment of VP2 protein sequences ordered by time was used to detect sustained versus sporadic changes in the protein relative to the ancestral sequence. Several changes, including T139M/A, I144V/T, and Y169H, occurred in multiple HCMC cluster 1 viruses with a much higher frequency of changes in the internal region of the protein (Fig. 6). It was previously noted that changes in this region of VP2 (VP1-interacting domain [VP_ID]) were associated with changes in VP1 (48). Both MEME and FUBAR identified VP2 codon 144 (marked with a red asterisk in Fig. 6) as being under positive selection.

**FIG 7:**
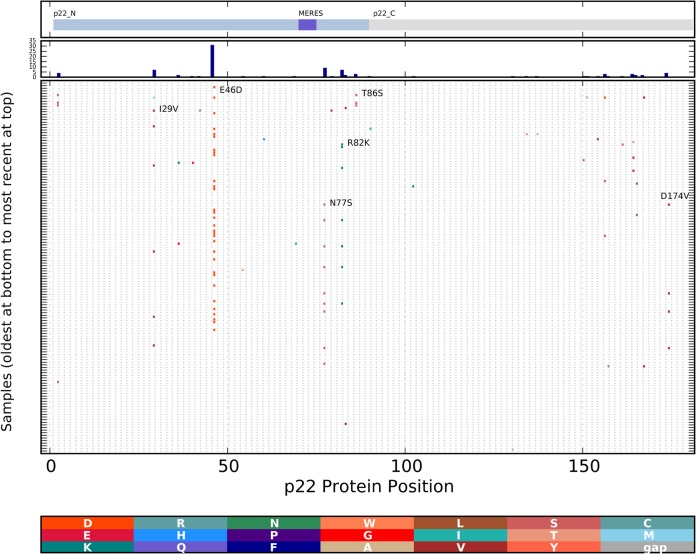
Changes in cluster 1 p22 (NS4) proteins over time. Protein changes were analyzed and are depicted as described in the legend to Fig. 5. The functional domains of the p22 (NS4) protein, including the MERES domain, are marked at the top. The histogram in the second panel from the top shows the total number of changes at each position.

**FIG 8:**
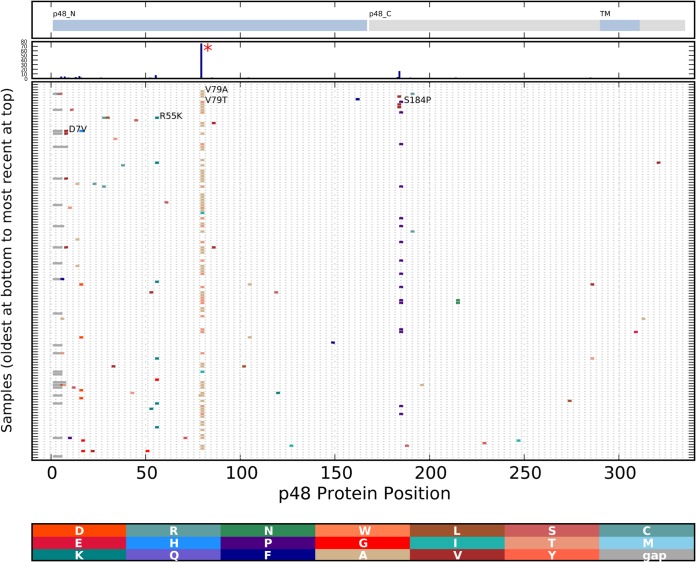
Changes in cluster 1 p48 (NS1-2) proteins over time. Protein changes were analyzed and are depicted as described in the legend to Fig. 5. The functional domains of the p48 (NS1-2) protein, including the transmembrane (TM) domain, are marked at the top. The histogram in the second panel from the top shows the total number of changes at each position. A codon found to be under positive selection by the MEME or FUBAR is indicated with a red asterisk.

The region encoding p22 (NS4) from the cluster 1 viruses showed higher evolutionary rates than the full genome (Table 5; Fig. 4). Analysis of all of the encoded p22 (NS4) molecules from cluster 1 (Fig. 7) showed amino acid differences from the ancestral sequence. Substitutions were observed in multiple isolates, suggesting neutral or positive selection (I29V, E46D, N77S, R82K, T86S, and D174V). Analysis of all of the encoded p48 (NS1-2) molecules from cluster 1 (Fig. 8) showed amino acid changes appearing in multiple isolates, suggesting neutral consequences with no constraints to limit change or positive selection (D7V, N15D, R55K, V79T [or V79A], and S184P). Both MEME and FUBAR identified p48 (NS1-2) codon 79 as being under positive selection (Table 6).

## DISCUSSION

Our work outlines a strategy for full-genome deep sequencing of norovirus directly from fecal specimens, and we have applied the strategy to characterize norovirus samples collected across a clinical spectrum of pediatric norovirus infections in HCMC, Vietnam. An essential component of the methods is a primer design algorithm that takes as input all of the available sequence data for a virus and quickly provides a set of functional primers. The flexible design of the primer design algorithm avoids a cumbersome alignment step in the process and facilitates regular updates with new sequence data. This is essential to avoid perpetuating a bias in the sequence data whereby sequences are obtained only if primers have functioned and primers are designed on antiquated data sets. The method showed a high success rate of full-genome sequencing of GII noroviruses, especially GII.4, which was predictable given that GII.4 genomes dominated the sequence data set used to design the primers. Future primer sets will be designed by using more targeted and updated genome sets and including more sequence data from other genogroups.

Results obtained by this method have provided a large set of norovirus genome sequences derived from longitudinal samples from one location. At the start of this study, 265 full norovirus genomes were available in the GenBank database; this study added an additional 112 genomes. The data allowed the estimation of evolutionary rates for several genotypes, for full genomes, as well as for subgenomic regions. The evolutionary pressures and the constraints to avoid change are not expected to be uniform across the virus genome. Selection pressures are likely to vary greatly, depending on the function of the encoded proteins, with enzymatic and structural regions more constrained then surface- and immune-exposed or spacer regions with less-well-defined functions. The ORF-specific substitution rates estimated for the three phylogenetic clusters show that the ORF1 regions exhibited evolutionary rates lower than those of the ORF2 (VP1) regions.

Previous studies have estimated that norovirus GII.4 and GII.3 VP1 capsid regions evolve at 5.1 × 10^−3^ to 5.8 × 10^−3^ substitution per site per year (49–51), while it was estimated that the GII.4 polymerase region evolve at 4.33 × 10^−3^ to 8.98 × 10^−3^ substitution per site per year, depending on the data set used (49). Our estimates based on HCMC data are consistent with these previously published values. The evolutionary rate determined for GII.4 cluster 1 was higher than the estimated rates for GII.4 cluster 4 and the GII.3 cluster 5 viruses, perhaps because of a greater number of cluster 1 infections per unit of time and thus a greater number of replication events. Alternatively, the three virus genotypes might have intrinsically different replication properties, polymerase fidelity, or immune selection pressure that result in the differing rates.

The norovirus sequence data obtained from this study allowed an analysis of the evolutionary patterns of the second viral capsid protein VP2. The high evolutionary rates reported here (cluster 5, 7.38 × 10^−3^ substitution per site per year; cluster 1, 8.99 × 10^−3^ substitution per site per year) have not been observed previously, as this region was seldom included in previous sequencing projects. The structure of VP2 is not defined, although there is evidence that the protein is interior to the VP1 shell and may be important for assembly of the VP1 structure (52). The protein is moderately basic, and the C-terminal half of the protein is rich in serine and threonine residues (providing possible phosphorylation sites) and proline residues (perhaps accounting for the inability to define the structure of this protein). Evidence that changes in VP2 accompany changes in VP1 has been presented (48). Recently, MNV VP2 has been shown to influence the host immune response to the virus, with MNV1 VP2 interfering with antigen-presenting cell function and MNV3 VP2 promoting the response (53). These observations identify a possible site of virus-host interaction that could be a source of selective pressure. The evolutionary rates of the VP2-encoding regions were found here to be much higher than that of the well-studied norovirus VP1 region, and the higher rates are consistent with a less constrained protein product, stronger selection pressures, or both. Positive-selection analysis across the VP2 region identified position 144 as being under selection; this region of the protein was previously found to be involved in interactions of VP2 with VP1 (52). A high evolutionary rate in a virus capsid protein suggests a region of the virion experiencing immune selection. Vaccine development efforts should take this accelerated rate of change into consideration when selecting components for a vaccine.

Humoral immunity to norovirus (at least GII.4) may involve blockade antibodies that bind and block the VP1 residues required for binding to HBGAs (16, 54, 55). The correlation of high-titer blockade antibodies with protection from gastroenteritis in challenge studies (29) and the frequent evolution of these sites (blockade epitopes A, D, and E) suggest that these amino acid residues may be frequent targets of immune selection (55). Blockade epitope D may be directly involved in HBGA binding (16, 54). Our observation of changes in VP1 position 298 epitope A, position 393 epitope D, and position 412 epitope E supports these previous conclusions. Several additional changes were located outside the blockade epitopes (S78G, S174P, G340E, and T502N, Fig. 5). Further studies should investigate whether these are founder effect changes of neutral consequence or if they provide an advantage for the virus.

Similar mean evolutionary rates for full genomes were found in clusters 1, 4, and 5, with 95% confidence interval ranges largely overlapping. One might expect a higher evolutionary rate for GII.4 viruses than for GII.3 viruses if the 10-fold higher detection frequency than GII.3 viruses directly reflects the community prevalence of these two infections. The similar full-genome rates suggest that either the number of active infections is not a large factor in the rate or that the less frequently diagnosed GII.3 infection is as frequent in the population as GII.4 but does not appear as frequently in clinics.

The ORF1-encoded p22 (NS4) regions showed a higher evolutionary rate than the full genome, and p48 (NS1-2) codon 79 was found to be under positive selection. The function of p22 (NS4) is not known, but the protein has been observed to localize to the Golgi compartment/endoplasmic reticulum (ER) and influence the host secretory pathway with a centrally located MERES (mimic of an ER export signal) motif required for localization (56, 57). The function of p48 (NS1-2) in norovirus infection is also largely unexplored, although the protein is reported to localize to vesicles and has been proposed to influence protein trafficking (58). The evidence that these viral proteins interact with host proteins, combined with the higher evolutionary rate or positive selection described here, suggests that these proteins may interact with host restriction factors. Alternatively, these regions with higher rates of change could encode proteins with no constraint. Further studies are needed to clarify this.

Extensive work has been done with the feline calicivirus and MNV models to elucidate the roles and interactions of the nonstructural (NS1-7) and structural (VP1 and VP2) proteins in the regulation of virus replication and infectivity, as comprehensively reviewed in reference 1. However, functional profiling of human norovirus is not yet possible because of the lack of tissue culture and animal models for human norovirus replication. The full-genome sequences of human norovirus available from this study provide valuable data on the spectrum of changes in the viral proteins allowed by the virus while awaiting alternative models for functional experiments.

This study has provided a description of norovirus evolution rates across HCMC over a 2.5-year period for the full genome, as well as for subgenomic regions, of the virus. We reveal for the first time a higher evolutionary rate in three regions of the genome (VP2, p22 [NS4], and p48 [NS1-2]) and provide evidence of positive selection in two coding regions (VP2 and p48 [NS1-2]). We suggest that these regions should be monitored for interactions with the host that might be a source of selective pressure. Finally, we believe that this study and the methods we have described will provide a useful template for community-wide studies of the full-genome evolution of many RNA virus pathogens.
